# Tracheotomy Before Total Laryngectomy as a Risk Factor for Stoma Recurrence

**DOI:** 10.3390/diagnostics16101552

**Published:** 2026-05-20

**Authors:** Andreza de Jesus Prates, Otávio Alberto Curioni, Daniel Marin Ramos, Bruno Pelinson Fogaça Duarte, Rogério Aparecido Dedivitis

**Affiliations:** 1 Department of Physiopathology, University of São Paulo School of Medicine, Sao Paulo 01246-903, Brazil; andrezajp.bh@gmail.com; 2 Department of Head and Neck Surgery and Otolaryngology, Heliópolis Hospital, Sao Paulo 04231-030, Brazil; otaviocurioniheliopolis@gmail.com; 3 Department of Head and Neck Surgery, Instituto do Câncer do Estado de São Paulo, University of São Paulo School of Medicine, Sao Paulo 01246-000, Brazil; danimramos@gmail.com; 4 Metropolitan University of Santos School of Medicine, Santos 11050-250, Brazil; bruno_duarte72@yahoo.com.br; 5 Department of Head and Neck Surgery, University of São Paulo School of Medicine, Sao Paulo 01246-903, Brazil

**Keywords:** laryngeal cancer, carcinoma, squamous cell, laryngectomy, tracheotomy, neoplasm recurrence

## Abstract

**Background/Objectives:** Laryngeal cancer has a high incidence and mortality rate, especially in advanced stages. Stoma recurrence after total laryngectomy is rare but lethal, and its risk factors remain controversial. We aimed to assess whether the presence of a tracheotomy performed prior to surgical treatment for advanced laryngeal cancer represents a risk factor for recurrence of the disease in the stoma and interferes with patient survival. **Methods:** A retrospective study was carried out in 138 patients who underwent total laryngectomy. Clinical-pathological variables were related to the presence of a preoperative tracheotomy. **Results:** Fifty-two patients without and 86 patients with preoperative tracheotomy were studied. There was a significant predominance of transglottic tumors (*p* = 0.02) and no patients with early-stage disease (T1 and T2, *p* = 0.002) in the tracheotomy group. There was no statistically significant difference in stoma recurrence (*p* = 0.53). On multivariate analysis, the risk of stoma recurrence was not significantly associated with the presence of a preoperative tracheotomy. **Conclusions:** The presence of a tracheotomy prior to total laryngectomy was not a risk factor for disease recurrence in the stoma or for reduced survival in the group.

## 1. Introduction

According to data published by GLOBOCAN, in 2018, the number of new cases of laryngeal cancer worldwide was 177,422, with mortality of 94,771 people [1]. Survival rates vary between 60% and 83%, according to the subsite of the disease, for early stages, with survival falling to 30% in advanced stages [2]. Among the recommended treatments are radiotherapy, radiotherapy associated with chemotherapy, or surgical treatment (with or without adjuvant treatment), depending on the stage of the disease and the patient’s clinical condition and choice [2,3]. In locally advanced laryngeal tumors classified as T4, the treatment with the best therapeutic outcome is total laryngectomy followed by adjuvant treatment.

One of the most dramatic situations in the follow-up of patients undergoing total laryngectomy is the recurrence of disease at the stoma during clinical follow-up. Such recurrences are defined as a diffuse infiltration of neoplastic tissue at the junction of the amputated trachea and the skin [4]. Preoperative tracheotomy was the most frequently reported risk factor for stoma recurrence in the literature, leading to the hypothesis that the tracheotomy tract allowed implanted tumor cells from the larynx to multiply and spread to the peritracheal space, resulting in stoma recurrence [5]. Stoma recurrences show an overall mortality rate of over 90%, with 80% of patients dying within the first two years after detection. Asphyxiation due to airway obstruction or erosion of the great vessels in the neck are the main causes of death. Even in patients who undergo salvage surgery, since the response to radiotherapy is limited, survival does not exceed 25% [6].

Determining risk factors is therefore highly relevant. Most authors consider advanced stage, central level lymph node involvement, subglottic tumor location, and lack of adjuvant radiotherapy as risk factors for stoma recurrence. However, it has not yet been clarified whether the presence of a tracheotomy prior to surgical treatment is a risk factor [6].

The aim of this study was to assess whether the presence of a tracheotomy prior to total laryngectomy as the primary surgical treatment is a risk factor for stoma recurrence and reduced survival in patients diagnosed with advanced laryngeal cancer.

## 2. Materials and Methods

This study was approved by the Institutional Review Board, approval number 4.709.121.

This is a retrospective multicentric study in which 335 medical records of consecutive patients who underwent total laryngectomy between January 2008 and December 2020 were reviewed. A total of 138 patients were selected who met all the analyzed variables, as well as the inclusion and exclusion criteria, from the Department of Head and Neck Surgery of two tertiary oncological care centers. All patients were treated according to the same protocol.

The following inclusion criteria were considered: histopathological diagnosis of squamous cell carcinoma; patients who underwent resection of the primary laryngeal tumor by total laryngectomy as upfront treatment; and a minimum postoperative follow-up period of 18 months. The following exclusion criteria were considered: primary treatment at another institution; patients previously treated with radiotherapy or chemotherapy. Patients with incomplete data for any of the analyzed variables were excluded from the study. Clinical suspicion was raised upon detection of a new mass or diffuse infiltration at the tracheostoma during routine follow-up. Stomal recurrence was defined as histopathologically confirmed neoplastic recurrence at the junction of the resected trachea and skin, identified during clinical and radiological follow-up and confirmed by biopsy. The outcomes evaluated were recurrence rates and overall survival.

Statistical analysis. The distribution of clinical and demographic characteristics between the groups was assessed using the chi-square test and Fisher’s exact test with a 95% confidence interval. The Kaplan–Meier method was used for recurrence and morbidity rates, with comparisons made using the log-rank test. Survival analysis was conducted using Cox regression, adjusted by the stepwise forward selection method.

## 3. Results

**Figure 1:**
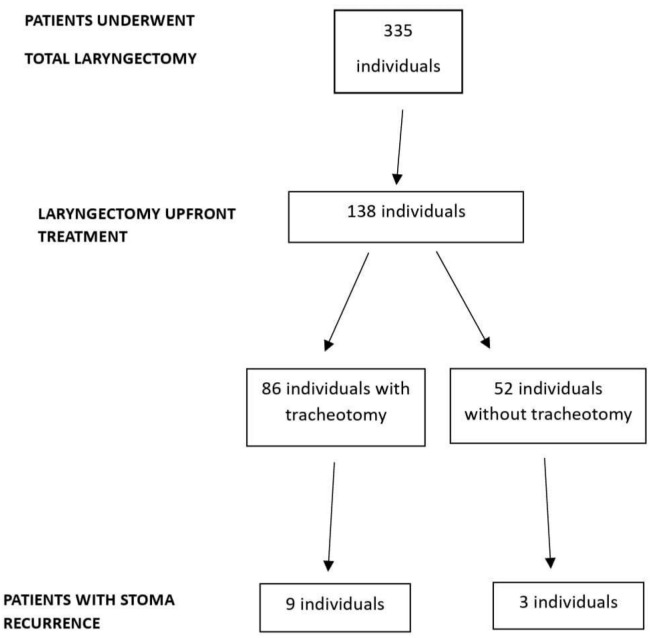
Flow diagram participant selection process.

We reviewed the medical records of 138 patients who underwent total laryngectomy as upfront treatment for laryngeal squamous cell carcinoma between 2008 and 2020, of whom 86 underwent tracheotomy before laryngectomy (Figure 1).

Most patients were male (87%), aged between 44 and 78 years at the time of diagnosis—Table 1. Smoking and alcohol consumption were verified in 95.65% and 80.43% of patients, respectively; 46.38% had glottic tumors, 29.71% had transglottic tumors, and 23.91% had supraglottic tumors at diagnosis.

With regard to the clinical stage of the primary tumor, 94 patients were staged as T4 and 40 as T3. After surgery, 77.54% of the patients underwent adjuvant chemoradiotherapy—Table 2.

The average follow-up time was 17.7 months (±26.6 meses); the median was 40.9 with an interquartile range of 21.1 to 56.0. The average duration of previous tracheotomy was 2.4 months. There were 12 stoma recurrences (8.69%) and 18 locoregional recurrences (13.04%). Of the stoma recurrences, nine occurred in patients who had undergone preoperative tracheotomy—Table 3.

### 3.1. Comparison of Groups in Relation to the Procedure Performed

All patients underwent total laryngectomy. Bilateral level VI neck dissection was significantly more frequent in patients with a previous tracheotomy (*p* = 0.042)—Table 2.

### 3.2. Comparison of Groups in Relation to Anatomopathological Results

There was a significant association between the presence of a previous tracheotomy and the presence of a transglottic neoplasm (*p* = 0.013). T4 stage was more frequent among patients with previous tracheotomy. There was no statistical difference in lymph node stage between the groups (*p* = 0.582) or in differentiation grade (*p* = 0.185), lymph node involvement (*p* = 0.497), involvement of surgical margins (*p* = 0.396), perineural invasion (*p* = 0.357), or angiolymphatic invasion (*p* = 0.471)—Table 2.

### 3.3. Comparison Between Groups According to Recurrence

The incidence of stoma recurrence was 11% in the tracheotomy group and 6.3% in the group without tracheotomy, with no statistical significance (*p* = 0.336)—Table 3.

Stoma recurrence was also evaluated as a factor strongly associated with a shorter time to death of 19.7 months (±5.8 months), *p* < 0.001. Other variables, such as the presence of a tracheotomy, did not show significant differences in survival—Table 4.

### 3.4. Evaluation of Tracheotomy as a Factor Impacting on Stoma Recurrence

When comparing the factors between patients who underwent tracheotomy and those who did not prior to total laryngectomy, no statistically significant difference was observed in the probability of stoma-recurrence-free survival (*p* = 0.357).

Multivariate overall survival analysis revealed that individuals with stoma recurrence have a significantly higher risk of mortality (HR = 9.601; 95% CI: 3.228–28.553; *p* < 0.001). In addition, individuals with involvement of sites other than the subglottis and transglottis also showed an increased risk of death (HR = 3.820; 95% CI: 1.088–13.406; *p* = 0.036). Individuals in the tracheotomy group did not show significant differences in survival compared to those without tracheotomy (HR = 1.235; 95% CI: 0.453–3.367; *p* = 0.681). The proposed model was also adjusted according to age, smoking load, and T staging. In the analysis of stoma recurrence, none of the evaluated factors showed a significant association with recurrence—Table 4.

## 4. Discussion

Stoma recurrence in patients undergoing total laryngectomy for laryngeal squamous cell carcinoma is one of the most feared diagnoses in oncological follow-up in head and neck surgery, given its poor prognosis and a mortality rate approaching 80% within the first two years [6]. This study associated stoma recurrence with a shorter survival time, with a mean time to death of 19.7 months (±5.8 months), with *p* < 0.001 compared to patients without recurrence.

Recurrence at the stoma has been associated with several possible mechanisms of tumor dissemination, including implantation of tumor cells in stomal tissues due to preoperative tracheotomy, diffusion of cancer cells through pretracheal tissues or the thyroid, and paratracheal lymphatic metastasis related to incomplete paratracheal dissection during laryngectomy [6]. There are several risk factors that could increase the chances of local recurrence in these patients, such as advanced tumor staging, disease in the central compartment of the neck, tumor involvement of the subglottis, and lack of adjuvant treatment [4,6]. The use of tracheotomy in the preoperative period before total laryngectomy is still a factor under study.

This study found a significant association between previous tracheotomy and transglottic neoplasms, with a higher number of these patients in the group with previous tracheotomy (*p* = 0.013). There was also a higher number of cases with T4 stage disease (*p* = 0.002) among patients with a previous tracheotomy. The higher indication for tracheotomy before total laryngectomy in association with advanced tumors is due to possible airway obstruction. A meta-analysis [4] found a higher number of stoma recurrences in patients with preoperative tracheotomy associated with advanced staging, with a high probability that the presence of occult peritracheal metastases not resected during total laryngectomy was the cause of local recurrences [4,7].

The average duration of tracheotomy prior to total laryngectomy was 2.4 months (±2.2). The studies that have so far attempted to associate the duration of preoperative tracheotomy with recurrence have done so over short periods of time. One study attempted to associate it with a duration of less than 21 days [8] and another with 24 h [9]. However, the results were not significant. The patients had a long preoperative tracheotomy duration in our study, so the assessment of tracheotomy duration did not show different intervals for comparison between groups.

This study showed no difference between the groups with and without a previous tracheotomy regarding the incidence of stoma recurrence (*p* = 0.533), as reported in other studies [6,7,9,10]. Only one retrospective study showed a statistically significant result pointing to a higher incidence of stoma recurrence in previously tracheotomized patients (*p* = 0.001), with a sample of 589 patients and a total of 48 stoma recurrences, showing a significant association [5]. One meta-analysis identified preoperative tracheotomy as a high-risk factor for stomal recurrence (RR = 1.959, *p* < 0.001), contrasting with this study [6,7].

Stoma recurrences can occur due to the presence of disease in lymph nodes at level VI that were not dissected during total laryngectomy due to technical difficulties in the presence of a preoperative tracheotomy [4,5,6,11]. It has been suggested that level VI neck dissection be carried out in patients with a previous tracheotomy to reduce the likelihood of local recurrence [5,6,10]. In this study, bilateral level VI dissection was significantly more frequent in patients with previous tracheotomy when compared to those without previous tracheotomy (*p* = 0.042), which could justify the low recurrence rates observed in this study.

In this study, patients had an overall survival rate of 85% at three years and 82.4% at five years. The presence of a tumor in the subglottis and advanced staging (T4) showed a reduction in mean survival. However, previous tracheotomy was not found to be a significant factor affecting survival. This finding was in line with one study, which also showed no difference in survival between groups with and without a previous tracheotomy [8].

The main factors associated with stoma recurrence are the presence of a tumor in the subglottis and advanced staging (T4). Tumors involving the subglottis tend to infiltrate the tracheal mucosa, and their lymphatic drainage includes central lymph nodes, increasing the risk of metastasis to level VI [4]. Infiltration of the thyroid cartilage through the cricothyroid membrane in advanced tumors predisposes to metastatic dissemination to level VI due to lymphatic drainage to Delphian and pretracheal lymph nodes [10]. The pathophysiological mechanisms of stoma recurrence are the spread of disease through the trachea and progression of disease in central lymph nodes [4]. Central neck dissection has been consolidated over the years [5,10] in patients with tracheotomy prior to total laryngectomy as a factor that interferes with the risk of stoma recurrence and the survival of these patients.

It is difficult to define independent risk factors for stoma recurrence due to the concomitance of these factors in individuals with the disease. An association between tracheotomy, tumor staging, and tumor location was suggested, since the patients who underwent emergency tracheotomy presented with more advanced-stage disease and/or tumor spread to the subglottis, which would be the cause of the airway obstruction. These factors together increase the risk of metastatic disease at level VI, which is responsible for stomal recurrence [12].

The multivariate survival analysis showed that stoma recurrence represents a higher risk of patient mortality; however, there was no significantly increased risk of stoma recurrence for patients with previous tracheotomy, as reported in other previous studies [5,6,7,8,9]. Nevertheless, a Chinese study showed a significant result in its multivariate analysis, associating the factors of primary tumor location, advanced staging, and compromised surgical margins with a greater chance of stoma recurrence [5]. Contrasting with our findings, a 2024 study of 119 laryngectomized patients provides important new evidence showing that preoperative tracheostomy is independently associated with thyroid gland invasion (OR 3.13) and demonstrates adverse survival outcomes, with lower 5-year disease-free survival (38% vs. 57%, *p* = 0.01) and overall survival (40% vs. 56%, *p* = 0.03) [13]. A 2026 meta-analysis is highly relevant, as it includes 4339 patients and provides the most comprehensive recent evidence on the impact of preoperative tracheotomy on stomal recurrence. Key findings include pooled local/peristomal recurrence rates of 17.9% with preoperative tracheotomy versus 7.0% without, and demonstrate that postoperative radiotherapy reduces recurrence risk in the tracheotomy group (12.5% vs. 34.8% without PORT) [14].

The role of preoperative tracheotomy may differ substantially according to the extent of laryngeal resection and the underlying oncological and functional status of the patient. In total laryngectomy, tracheotomy is often associated with advanced-stage disease, airway compromise, bulky tumors, aspiration risk, and poorer baseline clinical conditions, thereby functioning as a marker of disease severity. Conversely, in partial laryngectomy, including transoral approaches [15], where organ preservation and postoperative functional recovery are primary objectives, preoperative tracheotomy may exert a greater impact on local contamination, peristomal inflammation, bacterial colonization, and changes in laryngeal biomechanics, potentially influencing wound healing and functional outcomes.

Previous tracheotomy has traditionally been considered a possible risk factor for stomal recurrence after total laryngectomy. This association remains difficult to interpret, since tracheotomy is usually required in patients with airway compromise caused by more advanced disease. In the present study, previous tracheotomy was not associated with recurrence at the tracheotomy site. Nevertheless, patients undergoing preoperative tracheotomy had significantly more transglottic tumors and more advanced T stage, demonstrating an important baseline imbalance. Therefore, tracheotomy may represent a marker of extensive local disease rather than an independent causal factor. The need for airway intervention frequently reflects bulky tumors, subglottic extension, and impaired laryngeal mobility, all of which are themselves associated with poorer oncologic outcomes. Accordingly, the absence of a significant association should be interpreted cautiously. Overall, our findings suggest that previous tracheotomy is more likely a surrogate marker of advanced tumor burden than an independent predictor of stomal recurrence.

One factor that determines the difficulty in conducting similar studies is the decreasing number of upfront total laryngectomies. Most patients undergo radiotherapy and chemotherapy as neoadjuvant treatments, and laryngectomy surgery has been indicated for salvage treatment, which would represent another avenue of study [4,6,12]. In addition, stoma recurrence is an outcome with a low occurrence rate, making analysis difficult.

This study has some limitations. First, it was retrospective. Another factor is the lack of further pathological details regarding compromised central compartment lymph nodes and thyroid infiltration, which could be associated with recurrence in the stoma region, as suggested in previous studies [5,6,10]. Loss to follow-up among oncologic patients treated in public hospitals in developing countries remains a significant challenge and may adversely affect survival outcomes, treatment adherence, and post-therapeutic surveillance. This phenomenon is multifactorial, reflecting socioeconomic vulnerability, limited health literacy, transportation difficulties, long travel distances to tertiary referral centers, and indirect treatment-related costs despite publicly funded care. Thus, loss of follow-up can introduce biases in retrospective analyses of cohorts derived from public healthcare institutions in low- and middle-income countries.

The small number of stomal recurrences observed in this series (n = 12) represents an important limitation of the multivariable Cox model. Including multiple covariates with few outcome events may lead to model instability, overfitting, inflated variance estimates, and unreliable hazard ratios. Although variables were selected based on clinical relevance, the low event-per-variable ratio requires cautious interpretation of adjusted estimates, which should be considered exploratory rather than definitive. Therefore, the absence of statistically significant associations does not necessarily indicate lack of effect, and residual confounding cannot be excluded, particularly in this retrospective cohort with baseline imbalances between groups.

## 5. Conclusions

The presence of tracheotomy before total laryngectomy is associated with cases of transglottic tumors and advanced-stage disease. There was no significant association between recurrence and duration of tracheotomy. The presence of previous tracheotomy was not associated with survival. The presence of stoma recurrence increases the risk of death in patients, but there was no significant increase in the risk of stoma recurrence in the preoperative tracheotomy group.

## Figures and Tables

**Table 1:** Distribution of sociodemographic characteristics and health habits among individuals with and without preoperative tracheotomy.

	Groups				*p*-Value
	Not Tracheotomy		Tracheotomy Preoperative		
	n	% (IC95%)	n	% (IC95%)	
**Sex**					
Female	7	13.5 (6.2–24.6)	11	12.8 (7–21)	0.552
Male	45	86.5 (75.4–93.8)	75	87.2 (79–93)	
**Age (years)**					
≤55	14	26.9 (16.3–40)	16	18.6 (11.5–27.8)	0.336
56–60	19	36.5 (24.5–50.1)	29	33.7 (24.4–44.1)	
61–65	7	13.5 (6.2–24.6)	22	25.6 (17.3–35.5)	
>65	12	23.1 (13.3–35.8)	19	22.1 (14.3–31.7)	
**Smokers**	51	98.1 (91.4–99.8)	81	94.2 (87.7–97.7)	0.409
**Smoking**					
≤20	8	16 (7.9–27.9)	8	9.9 (4.8–17.8)	0.611
21–40	21	42 (29.1–55.8)	39	48.1 (37.5–58.9)	
≥41	21	42 (29.1–55.8)	34	42 (31.7–52.8)	
**Smoking cessation**	51	100	81	100	0.548
**Alcohol use**	41	78.8 (66.4- 88.2)	70	82.4 (73.2–89.3)	0.657
**Alcohol cessation**	37	90.2 (78.5–96.6)	65	87.8 (79.0–93.8)	0.769

*p*-value based on the chi-square test or Fischer’s exact test.

**Table 2:** Surgical and pathological data.

	Groups				*p*-Value
	Not Tracheotomy		Tracheotomy Preoperative		
	n	% (IC95%)	n	% (IC95%)	
**Tumor site**					
Glottic	27	51.9 (38.5–65.1)	37	43 (32.9–53.6)	0.200
Supraglotttic	15	28.8 (17.9–42.1)	20	23.3 (15.3–33)	0.466
Subglottic	6	11.5 (5–22.2)	0	0.0	0.020
Transglottic	9	17.3 (8.9–29.2)	32	37.2 (27.6–47.7)	0.013
**T-stage**					
1	3	5.8 (1.7–14.6)	0	0.0	0.002
2	2	3.8 (0.8–11.8)	0	0.0	
3	19	36.5 (24.5–50.1)	20	23.3 (15.3–33)	
4	28	53.8 (40.4–66.9)	66	76.7 (67–84.7)	
**N stage**					
N0	31	59.6 (46.1–72.1)	43	50 (39.6–60.4)	0.582
N1	3	5.8 (1.7–14.6)	11	12.8 (7–21)	
N2a	2	3.8 (0.8–11.8)	1	1.2 (0.1–5.3)	
N2b	6	11.5 (5–22.2)	12	14 (7.9–22.4)	
N2c	6	11.5 (5–22.2)	9	10.5 (5.3–18.2)	
N3a	0	0.0	0	0.0	
N3b	4	7.7 (2.7–17.3)	10	11.6 (6.1–19.6)	
**M stage**					
M0	52	100.0	86	100.0	
**Surgical**					
Total laryngectomy	52	100.0	86	100.00	
Hemithyroidectomy	6	11.5 (5–22.2)	7	8.1 (3.7–15.3)	0.555
Total thyroidectomy	1	1.9 (0.2–8.6)	10	11.6 (6.1–19.6)	0.052
**Neck dissection**					
Unilateral levels II–IV	7	13.5 (6.2–24.6)	11	12.8 (7–21)	0.999
Bilateral levels II–IV	20	38.5 (26.2–52)	33	38.4 (28.6–48.9)	0.999
Modified radical + contralateral II–IV	14	26.9 (16.3–40)	26	30.2 (21.3–40.5)	0.704
Bilateral modified radical	5	9.6 (3.8–19.8)	10	11.6 (6.1–19.6)	0.785
Classic radical + modified contralateral	0	0.0	2	2.3 (0.5–7.3)	0.527
Classic radical + contralateral II–IV	1	1.9 (0.2–8.6)	1	1.2 (0.1–5.3)	0.999
Unilateral level VI compartment	7	13.5 (6.2–24.6)	9	10.5 (5.3–18.2)	0.595
Bilateral level VI compartment	5	9.6 (3.8–19.8)	21	24.4 (16.3–34.2)	0.042
No neck dissection	3	5.8 (1.7–14.6)	0	0.0	0.052
Unilateral modified radical	2	3.8 (0.8–11.8)	2	2.3 (0.5–7.3)	0.632
**Differentiation**					
Well differentiated	10	19.2 (10.3–31.4)	29	33.7 (24.4–44.1)	0.185
Moderately differentiated	35	67.3 (53.9–78.9)	48	55.8 (45.3–66)	
Poorly differentiated	7	13.5 (6.2–24.6)	9	10.5 (5.3–18.2)	
**Metastatic lymph nodes**	21	42.9 (29.7–56.8)	42	49.4 (39–59.9)	0.497
**Involvement of surgical margins**	17	32.7 (21.1–46.1)	25	29.1 (20.3–39.2)	0.396
**Perineural invasion**	21	40.4 (27.9–53.9)	27	31.4 (22.3–41.7)	0.357
**Angiolymphatic invasion**	8	25.8 (13–42.9)	21	24.4 (16.3–34.2)	0.528
**Radiotherapy**	38	73.1 (60–83.7)	70	81.4 (72.2–88.5)	0.291
**Chemo-radiotherapy**	21	40.4 (27.9–53.9)	29	34.1 (24.7–44.6)	0.471

*p*-value based on the chi-square test or Fischer’s exact test.

**Table 3:** Comparison of incidence of recurrence, metastasis, and outcomes.

	Groups				*p*-Value
	Not Tracheotomy		Tracheotomy Preoperative		
	n	% (IC95%)	n	% (IC95%)	
Cervical recurrence	8	16.7 (8.2–29)	10	11.8 (6.2–19.9)	0.440
Stoma recurrence	3	6.3 (1.8–15.7)	9	11 (5.6–19.1)	0.533
Distant metastasis	9	39.1 (21.4–59.4)	16	18.8 (11.6–28.1)	0.053

*p*-value based on the chi-square test or Fischer’s exact test.

**Table 4:** Analysis of adjusted overall survival and stomal recurrence.

	B	SE	*p*-Value	HR	IC95%	
					Lower	Higher
**Model: overall survival**						
Preoperative tracheotomy	0.211	0.512	0.681	1.235	0.453	3.367
Age (years)	0.037	0.032	0.255	1.037	0.974	1.105
Smoking	0.006	0.011	0.570	1.006	0.985	1.029
Glottic + supraglottic sites	1.340	0.641	0.036	3.820	1.088	13.406
Stoma recurrence	2.262	0.556	<0.001	9.601	3.228	28.553
T4 Staging	1.340	0.754	0.075	3.819	0.872	16.734
**Model: Stoma recurrence**						
Preoperative tracheotomy	−0.565	0.683	0.409	0.568	0.149	2.170
Age (years)	0.015	0.04	0.715	1.015	0.938	1.097
Smoking	−0.011	0.015	0.484	0.99	0.961	1.019
Glottic + supraglottic sites	0.338	0.628	0.59	1.402	0.410	4.798
T4 Staging	0.682	0.789	0.388	1.977	0.421	9.285

HR: Hazard ratio; B: regression coefficient; SE: standard error; “Glottic + supraglottic sites” stratification allowed statistical difference.

## Data Availability

The original contributions presented in this study are included in the article. Further inquiries can be directed to the corresponding author.
